# Analysis of risk factors for tic disorders in children in Hubei region based on liquid chromatography-tandem mass spectrometry

**DOI:** 10.3389/fnins.2026.1811167

**Published:** 2026-05-04

**Authors:** Yingchan Hao, Ruoxi Ran, Li Cheng, Dan Lu

**Affiliations:** Department of Laboratory Medicine, Maternal and Child Health Hospital of Hubei Province, Tongji Medical College, Huazhong University of Science and Technology, Wuhan, China

**Keywords:** 25-hydroxyvitamin D, correlation analysis, liquid chromatography-tandem mass spectrometry, tic disorders, trace elements

## Abstract

**Objective:**

This study aims to investigate the correlation between trace element concentrations, 25-hydroxyvitamin D [25(OH)D] levels, and the severity of tic disorders (TD) in children from the Hubei region. Additionally, it seeks to explore the interrelationships among these monitored indicators to provide a reference for the clinical diagnosis and treatment of pediatric TD.

**Methods:**

A retrospective review was conducted on the medical records of 237 children diagnosed with TD (TD group) and 137 healthy controls, admitted to the Department of Neurology at Hubei Maternal and Child Health Care Hospital. The TD group was further divided into mild and moderate-to-severe subgroups based on the Yale Global Tic Severity Scale scores. General clinical data were collected, and serum trace element levels were measured using inductively coupled plasma mass spectrometry (ICP-MS), while serum 25(OH)D levels were quantified via liquid chromatography–tandem mass spectrometry (LC–MS/MS). Group comparisons, Spearman correlation analysis, and univariate/multivariate logistic regression analyses were performed.

**Results:**

The findings indicated that serum 25(OH)D levels were significantly lower in children with TD compared to controls (*p* < 0.001). Logistic regression analysis demonstrated that 25(OH)D was an independent protective factor against tic disorders.(*p* < 0.001). Compared to the control group, children with TD exhibited significantly lower levels of calcium and copper (*p* < 0.001), along with higher levels of iron and cadmium (*p* < 0.01). These differences were more pronounced in the subgroup of children older than 6 years. An age-stratified subgroup analysis revealed no significant differences in any other indicators except for cadmium between the TD and control groups among children aged 6 years or younger (*p* > 0.05). Spearman correlation analysis demonstrated that within the TD group, 25(OH)D levels had the most significant correlations with calcium and copper (*p* < 0.001). No statistically significant differences were observed in the levels of the 10 trace elements or 25(OH)D between the mild and moderate-to-severe TD subgroups (*p* > 0.05).

**Conclusion:**

Children with Tic Disorders in Hubei Province demonstrate a distinctive alteration in their micronutrient profile, primarily characterized by a deficiency in 25(OH)D, alongside reduced levels of calcium and copper, and elevated levels of iron and cadmium. This association is particularly pronounced in male children over the age of six. While 25(OH)D deficiency is identified as an independent risk factor for TD, its concentration does not significantly correlate with the severity of the disorder.

## 1 Introduction

Tic disorders (TD/Tics) are classified as neurodevelopmental disorders that typically manifest in childhood and are characterized by involuntary motor or vocal tics, with an estimated global prevalence of approximately 2.5% (Li et al., 2022). TD is often associated with a range of comorbid conditions, including attention-deficit/hyperactivity disorder, obsessive-compulsive disorder, anxiety disorders, and depressive disorders, which significantly impair the learning, social functioning, and overall quality of life of affected children (Baizabal-Carvallo et al., 2024). Although the etiology of TD is not yet fully understood, recent evidence suggests that its pathogenesis involves a complex interplay of neurotransmitter imbalances, genetic predispositions, immune dysfunctions, psychological stressors, and environmental influences (Breton et al., 2025). Current therapeutic approaches for TD primarily involve pharmacological interventions, including antipsychotics, alpha-agonists, and traditional Chinese medicine, as well as behavioral therapies (Pringsheim et al., 2019; Xiang et al., 2025). However, these treatments are often constrained by suboptimal efficacy and significant adverse effects. Consequently, the identification of quantifiable and modifiable early risk factors, along with the development of preventive strategies based on nutritional and environmental modifications, is of considerable clinical importance.

In recent years, the significance of vitamin D in the development and functioning of the nervous system has attracted considerable scholarly interest. Vitamin D plays a pivotal role in metabolism, immune function, inflammatory regulation, and neural development through its interaction with the vitamin D receptor (VDR), a member of the nuclear receptor family. Serum 25-hydroxyvitamin D [25(OH)D] is globally acknowledged as the most reliable biomarker for assessing vitamin D nutritional status (Grant et al., 2025). In addition to its role in regulating calcium and phosphorus metabolism, 25(OH)D influences central nervous system function by modulating the expression of neurotrophic factors, neurotransmitter release, and immune-inflammatory responses. Epidemiological studies indicate a potential association between vitamin D deficiency and various neuropsychiatric disorders, including autism spectrum disorder, attention-deficit/hyperactivity disorder, and tic disorders (Li et al., 2024). Trace elements, functioning as metalloenzymes, cofactors, or regulators of ion channels, are integral to neurotransmitter synthesis, myelination, and antioxidant defense mechanisms (Bjørklund et al., 2025). To date, limited research has examined the interactions among multiple trace elements and 25(OH)D in children with TD, as well as their contributions to the onset and severity of TD. Moreover, the micronutrient profile characteristics of children with TD in Hubei Province, a representative area in Central China, have not been systematically investigated.

This study utilizes liquid chromatography–tandem mass spectrometry (LC–MS/MS) and inductively coupled plasma mass spectrometry (ICP-MS) to systematically assess serum concentrations of 25(OH)D and 10 trace elements in children diagnosed with TD, as well as in healthy control subjects from Hubei Province. We investigated the associations between these biochemical indicators and the occurrence and severity of TD. By employing correlation heatmaps, multivariate regression analyses, and age-stratified examinations, we elucidated the potential mechanisms by which micronutrients influence TD and identified specific metabolic biomarkers. This research provides a scientific foundation for early clinical screening, risk assessment, and nutritional interventions.

## 2 Subjects and methods

### 2.1 Study subjects

A cohort of 237 pediatric patients diagnosed with tic disorders was admitted to the Department of Neurology at Hubei Maternal and Child Health Hospital from January 1, 2023, to October 31, 2025. These patients were designated as the TD group. Concurrently, a control group was constituted, comprising 137 healthy children matched for age, who were undergoing routine physical examinations in the Department of Child Health Care during the same timeframe. Tic severity was evaluated using the Yale Global Tic Severity Scale (YGTSS) (Haas et al., 2021). This scale is a clinician-rated instrument that assesses tics across five dimensions: number, frequency, intensity, complexity, and interference. In this study, the current severity score based on symptoms occurring during the past week was used to stratify the TD group, with a score of <25 defined as mild TD, and a score of ≥25 defined as moderate-to-severe TD. All YGTSS evaluations were conducted by trained patients on the day of enrollment. In addition, fasting venous blood samples were collected from all participants on the same day as the YGTSS assessment; therefore, there was no time interval between the scale evaluation and biomarker measurement. The inclusion criteria for the TD group were as follows: (1) a diagnosis of TD based on a comprehensive assessment aligned with the Diagnostic and Statistical Manual of Mental Disorders, Fifth Edition (DSM-5), characterized by one or more motor or vocal tics occurring multiple times daily; (2) age range of 3 to 16 years; (3) no prior history of TD treatment; and (4) the ability to communicate effectively, with requisite cognitive and reading skills. The exclusion criteria for this study included: (1) children with comorbid neuropsychiatric disorders; (2) a history of using dopamine receptor blockers, cocaine, or other medications for the management of Tourette’s Disorder; (3) individuals with underlying conditions such as severe respiratory, cardiovascular, hematological diseases, or circulatory system disorders; (4) incomplete clinical data; (5) patients with elevated anti-streptolysin O levels (>116 IU/mL) or abnormal ceruloplasmin levels. This study received approval from the Medical Ethics Committee of Hubei Maternal and Child Health Hospital (Approval No.2026–013-02).

### 2.2 Methods

#### 2.2.1 Quantification of serum vitamin D via liquid chromatography-tandem mass spectrometry (LC–MS/MS)

Fasting venous blood samples (3 mL) were obtained from all participants. Serum was isolated via centrifugation and preserved in an ultra-low temperature freezer at −80 °C until further analysis. Sample pretreatment involved protein precipitation followed by liquid–liquid extraction. The processed samples were subsequently injected into a liquid chromatography system, where they underwent chromatographic separation before introduction into a mass spectrometer (Thermo Scientific TSQ Altis MD, Thermo Fisher Scientific, China). Ionization occurred within the ion source, producing charged ions that were ultimately detected by the analyzer. Chromatographic separation was conducted using a Shim-pack Velox PFPP column (2.1 × 100 mm, 2.7 μm) maintained at 40 °C. The mobile phase comprised 0.1% formic acid aqueous solution and methanol, applied under gradient elution at a flow rate of 0.3 mL/min, with an injection volume of 5 μL. Mass spectrometry was operated in electrospray ionization positive ion mode with multiple reaction monitoring. Quantification was executed using an internal standard method and specialized mass spectrometry software. All procedures were meticulously performed in strict accordance with the kit instructions (H-bio, Jiangsu, China).

#### 2.2.2 Quantification of trace elements via inductively coupled plasma mass spectrometry (ICP-MS)

Fasting heparin-anticoagulated whole blood samples (2 mL each) were collected for analysis. Quantification of 10 trace elements-namely magnesium (Mg), calcium (Ca), manganese (Mn), iron (Fe), copper (Cu), zinc (Zn), arsenic (As), selenium (Se), cadmium (Cd), and lead (Pb)-was conducted using an inductively coupled plasma mass spectrometry (ICP-MS) instrument (Cin-ICP-QMS-I, Yixin Bochuang, China). The samples underwent digestion with nitric acid, followed by dilution to appropriate concentrations with deionized water. Rhodium was employed as an internal standard to correct for matrix interference and signal drift. Each sample was analyzed in triplicate, and the mean value was utilized for statistical evaluation. Calibration curves were constructed using certified standard solutions, exhibiting a linear range of 0.1–1,000 μg/L and correlation coefficients exceeding 0.995. Quality control was maintained through the use of low- and high-concentration quality control materials to ensure the precision and reliability of the analytical outcomes.

### 2.3 Statistical analysis

Statistical analyses were conducted utilizing SPSS software version 22.0 and GraphPad Prism software version 10.0. Measurement data adhering to a normal distribution and exhibiting homogeneity of variance are reported as the mean ± standard deviation (*χ_* ± *s*), with intergroup comparisons performed using the *t*-test. Categorical data are expressed as frequencies or percentages, and group comparisons were conducted using the chi-square test. For data not adhering to a normal distribution, results are presented as the median and interquartile range (M[Q25, Q75]), with the Mann–Whitney *U* test employed for intergroup comparisons. Variables demonstrating a significance level of *p* < 0.05 in the univariate analysis were incorporated into a multivariate logistic regression model to examine the associations between 25(OH)D, various trace elements, and tic disorders. Spearman’s rank correlation analysis was utilized to evaluate the correlations among trace elements and 25(OH)D in children with tic disorders. A *p*-value of less than 0.05 was considered indicative of statistical significance.

## 3 Results

### 3.1 Comparison of general characteristics

This study included a total of 237 children diagnosed with TD group who were admitted to the Department of Neurology at Hubei Maternal and Child Health Hospital between January 2023 and October 2025. Additionally, 137 healthy children (control group) who underwent physical examinations in the Department of Child Healthcare during the same period were included. The TD group consisted of 172 males (72.6%) and 65 females (27.4%), whereas the control group comprised 69 males (50.4%) and 68 females (49.6%). Statistical analyses were conducted using the Chi-square test and Mann–Whitney *U* test to compare sex and age distributions between the two groups, respectively. The analyses revealed statistically significant differences (*p* < 0.05); while residence distribution did not differ significantly between the two groups (*p* > 0.05), as detailed in Table 1.

**Table 1:** Comparison of general characteristics between the two groups.

Variables	TD (*n* = 237)	Control group (*n* = 137)	*χ2/Z*	*P*
Sex (%)				
Male	172 (72.6)	69 (50.4)	18.686	<0.001
Female	65 (27.4)	68 (49.6)		
Age	7.58 (5.83,9.00)	5.33 (4.00,8.00)	−5.812	<0.001
Residence (%)				
Urban	203 (85.7%)	112 (81.8%)	0.995	0.319
Rural	34 (14.3%)	25 (18.2%)		
YGTSS	27.61 ± 11.74	—		

### 3.2 Correlation analysis between trace elements and 25(OH)D levels in children with TD

**Figure 1:**
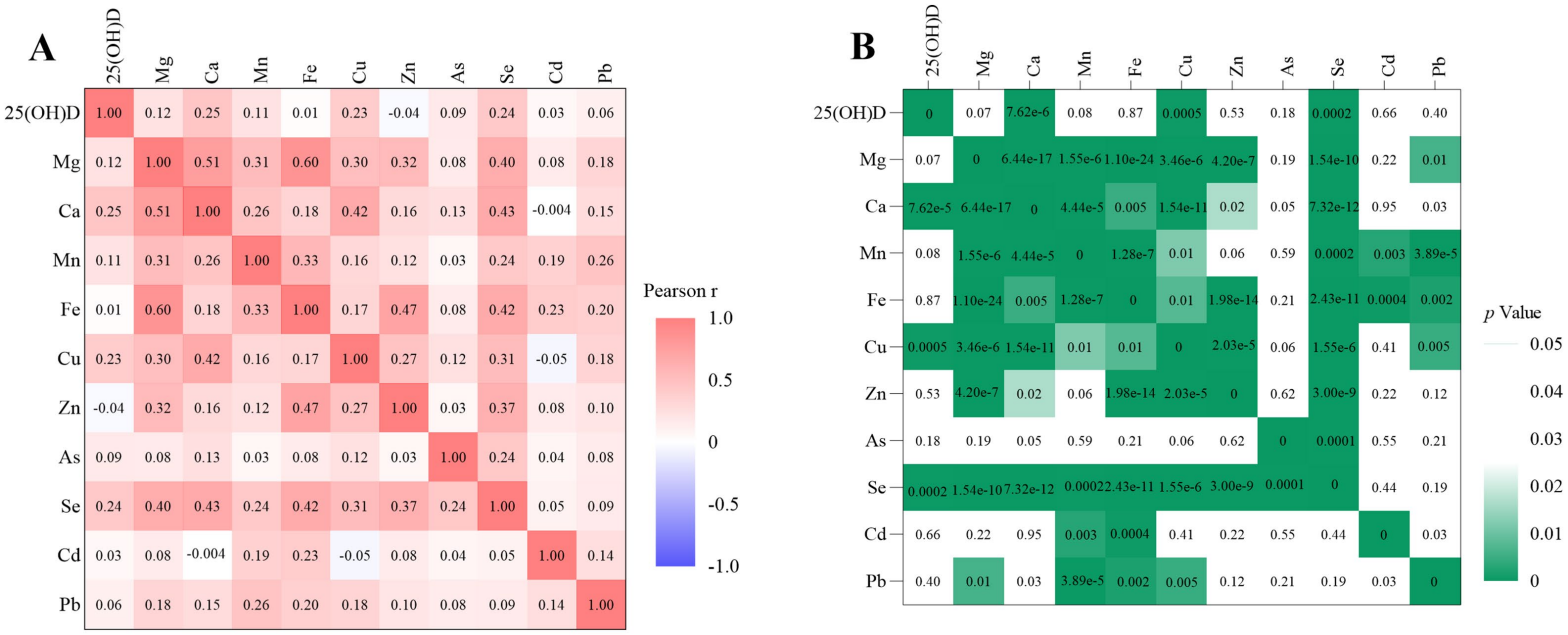
Correlation heatmap of trace elements and vitamins in children with TD. *r* = 1 indicates a complete positive correlation; *r* = −1 indicates a complete negative correlation; panel **(A)** represents the correlation coefficient *r* value; **(B)** represents the statistical *p*-value.

A Spearman correlation analysis was conducted on the trace element and 25(OH)D measurements from a cohort of 237 children diagnosed with TD. The analysis identified significant correlations both among the trace elements themselves and between the trace elements and 25(OH)D levels. Notably, positive correlations (*p* < 0.001) were observed between several trace elements: calcium was positively correlated with magnesium, manganese, copper, and selenium; manganese showed positive correlations with magnesium, iron, and lead; iron was positively correlated with zinc, chromium, and magnesium; copper exhibited positive correlations with magnesium and zinc; and selenium was positively correlated with magnesium, manganese, iron, copper, zinc, and arsenic. Furthermore, 25(OH)D demonstrated significant positive correlations with calcium, copper, and selenium (*p* < 0.001). These relationships are illustrated in Figure 1.

### 3.3 Comparison of trace element and 25(OH)D levels between the TD and control groups

**Figure 2:**
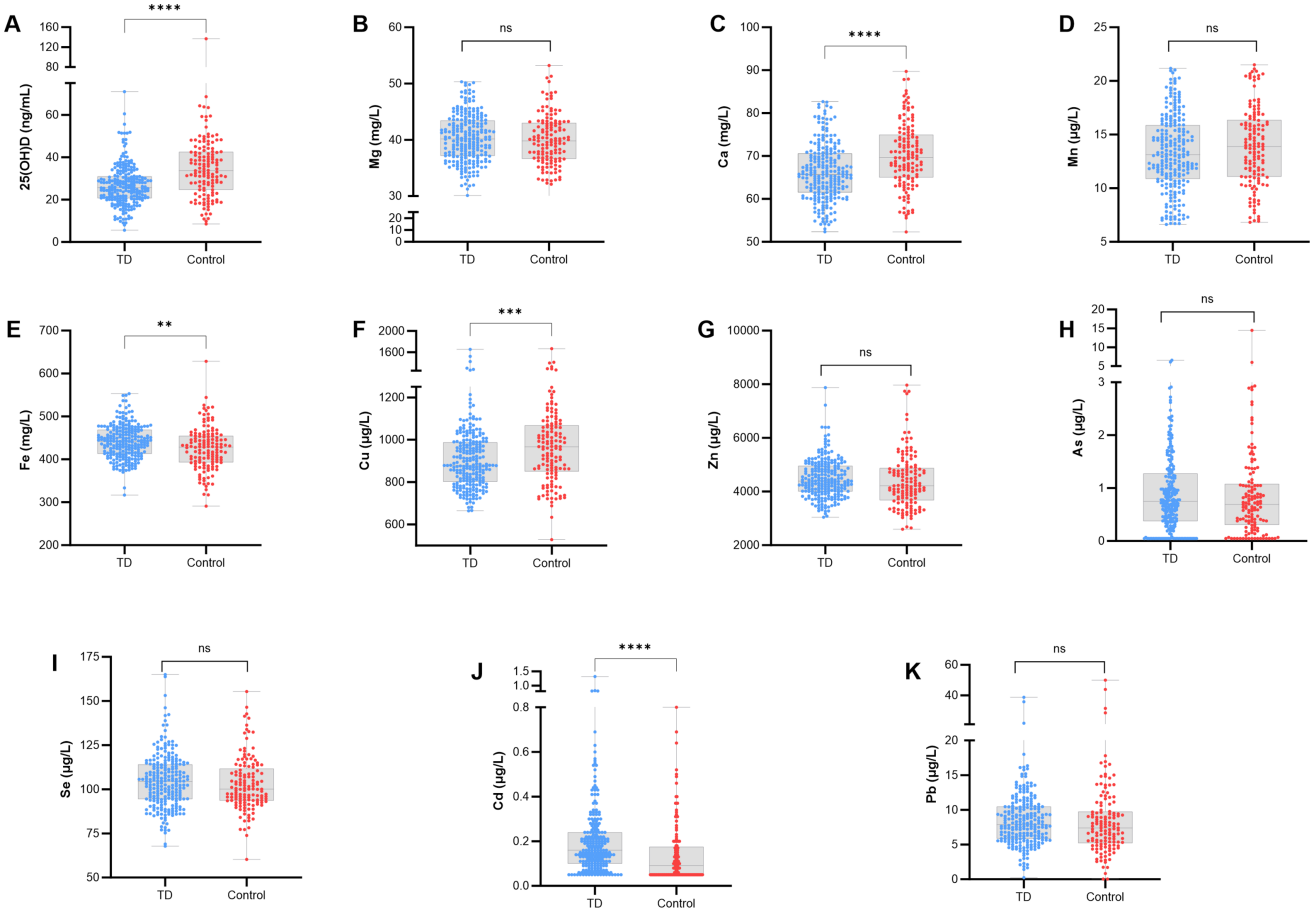
Comparison of the levels of trace elements and 25(OH)D between the two groups. **(A)** 25(OH)D; **(B)** Mg; **(C)** Ca; **(D)** Mn; **(E)** Fe; **(F)** Cu; **(G)** Zn; **(H)** As; **(I)** Se; **(J)** Cd; **(K)** Pb; ****p < 0.0001, ***p < 0.001, **p < 0.01; ns, not significant.

The analysis of trace elements and 25(OH)D levels indicated that the TD group had significantly elevated levels of Fe and Cd, alongside significantly reduced levels of Ca, Cu, and serum 25(OH)D compared to the control group (*p* < 0.0001). No statistically significant differences were found between the two groups concerning the levels of Mg, Mn, As, Zn, Se, or Pb (*p* > 0.05), as presented in Figure 2.

### 3.4 Analysis of independent effects of serum 25(OH)D on the risk of TD

Univariate and multivariate logistic regression analyses were performed to further evaluate whether serum 25(OH)D could serve as an independent factor for the occurrence of TD. In univariate analysis, each 1-unit increase in 25(OH)D level was associated with a 5.9% reduced risk of tic disorders (OR = 0.941, 95% CI: 0.921–0.961, *p* < 0.001). In Model 3, adjusted for both age and sex as confounding factors, the inverse association between 25(OH)D and tic disorders remained stable (OR = 0.943, 95%CI: 0.923–0.964, *p* < 0.001). Elevated 25(OH)D levels were significantly associated with a decreased risk of TD regardless of adjustment for age and sex, suggesting that 25(OH)D may act as a protective factor against TD (refer to Table 2).

**Table 2:** Analysis of independent effects of serum 25(OH)D on the risk of tic disorders.

Variable	OR	OR,95%CI	*P*
25(OH)D	0.941	0.921, 0.961	<0.001
Model 1	0.947	0.927, 0.967	<0.001
Model 2	0.938	0.918, 0.958	<0.001
Model 3	0.943	0.923, 0.964	<0.001

Model 1 adjusted for age; Model 2 adjusted for sex; Model 3 adjusted for both age and sex.

### 3.5 Analysis of risk factors for TD in children before and after school age (≤6 years and >6 years)

**Figure 3:**
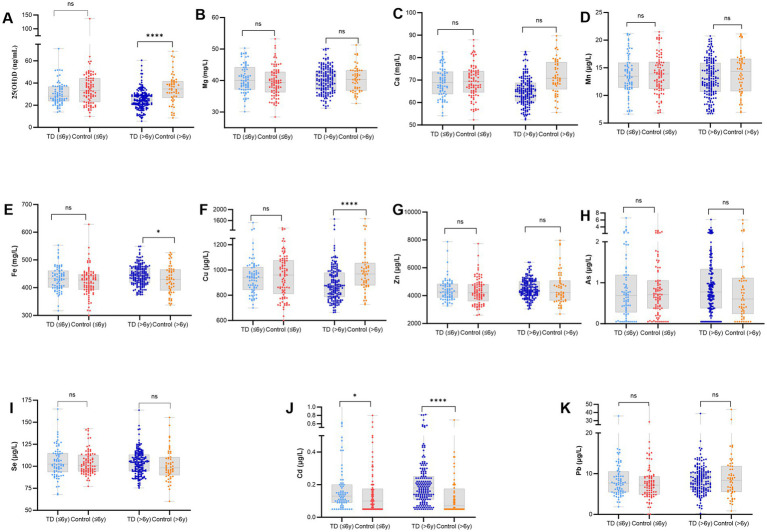
Comparison of the levels of trace elements and 25(OH)D between the two groups in the age subgroup >6 Years or ≤6 years. **(A)** 25(OH)D; **(B)** Mg; **(C)** Ca; **(D)** Mn; **(E)** Fe; **(F)** Cu; **(G)** Zn; **(H)** As; **(I)** Se; **(J)** Cd; **(K)** Pb; ****p < 0.0001, *p < 0.05; ns, not significant.

In the age category of ≤6 years, a statistically significant difference in Cd was observed between the two groups; however, no significant differences were identified in any other indicators. In contrast, for the age category >6 years, the TD group demonstrated significantly higher levels of Cd and significantly lower levels of Ca, Cu, and serum 25(OH)D in comparison to the control group, with these differences reaching statistical significance (*p* < 0.05), as detailed in Figure 3.

### 3.6 Risk factors for severe tic disorders

**Figure 4:**
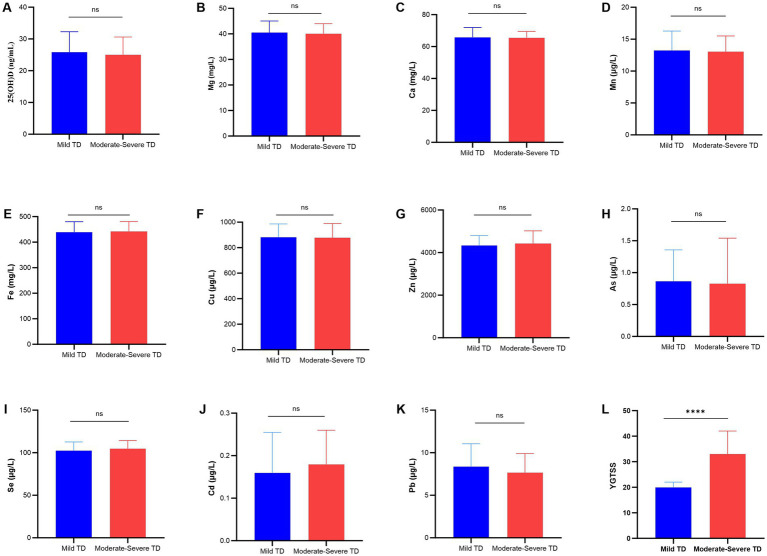
Comparison of trace element and 25(OH)D levels between the Mild and Moderate–Severe TD groups. **(A)** 25(OH)D; **(B)** Mg; **(C)** Ca; **(D)** Mn; **(E)** Fe; **(F)** Cu; **(G)** Zn; **(H)** As; **(I)** Se; **(J)** Cd; **(K)** Pb; **(L)** YGTSS; ****p < 0.0001; ns, not significant.

The moderate–severe TD group was significantly older than the mild TD group (*p* < 0.05). However, no statistically significant differences were observed in the levels of the 10 trace elements and 25(OH)D between the two groups (*p* > 0.05). The detailed data are presented in Figure 4.

### 3.7 Risk factor analysis of tic disorders in the TD group before and after school age (≤6 years and >6 years)

**Figure 5:**
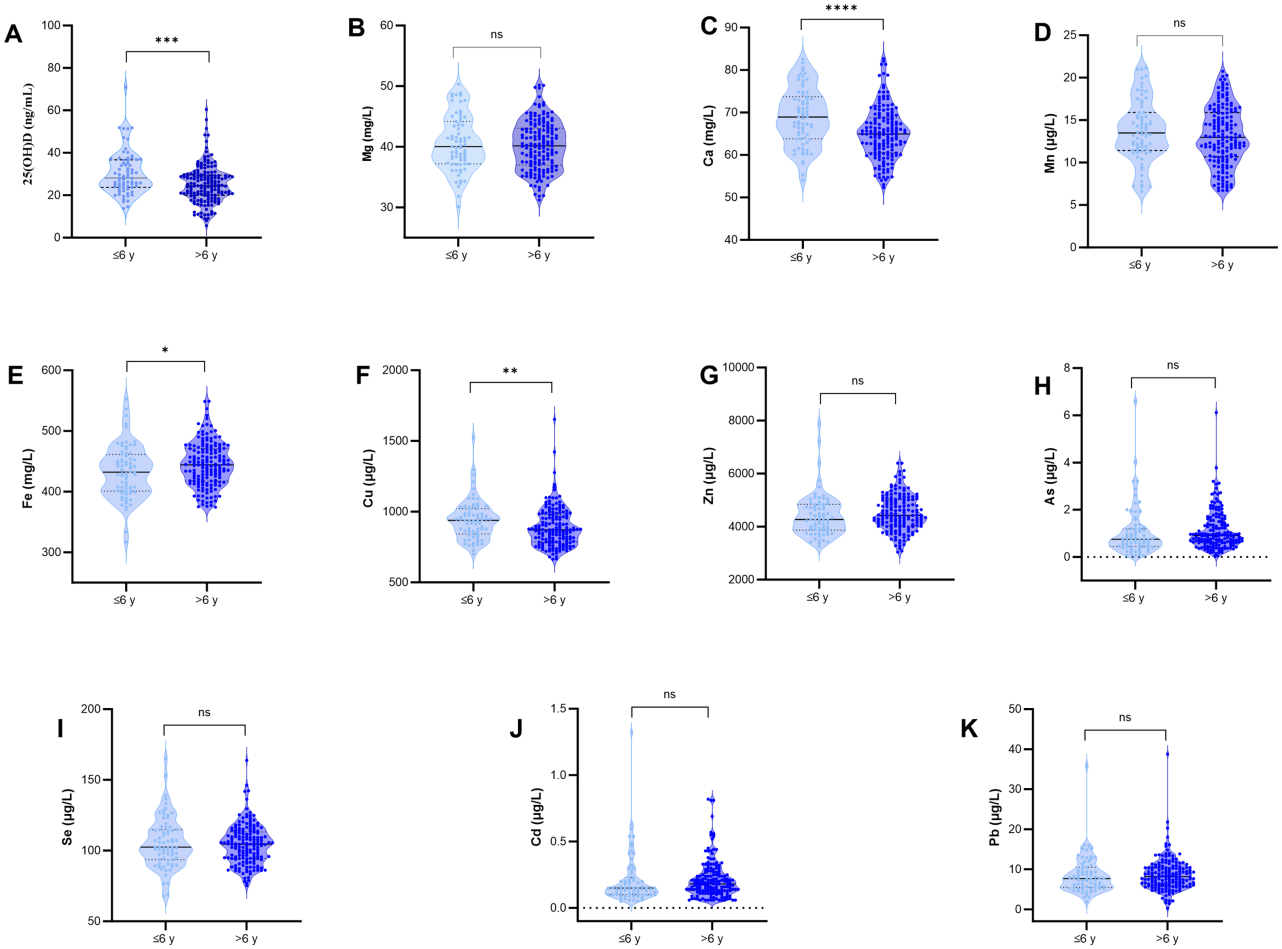
Comparison of trace elements and 25(OH)D levels in the TD Group across different age groups (≤6 years and >6 years). **(A)** 25(OH)D; **(B)** Mg; **(C)** Ca; **(D)** Mn; **(E)** Fe; **(F)** Cu; **(G)** Zn; **(H)** As; **(I)** Se; **(J)** Cd; **(K)** Pb; ****p < 0.0001, ***p < 0.001, **p < 0.01, *p < 0.05; ns, not significant.

The analysis revealed statistically significant differences in iron, calcium, copper, and serum 25(OH)D levels between the two age groups (*p* < 0.05). Children with tic disorders aged >6 years exhibited lower serum 25(OH)D levels, as well as reduced levels of the trace elements calcium and copper, compared to those aged ≤6 years. Refer to Figure 5 for detailed data.

## 4 Discussion

Tic disorder is a prevalent neurodevelopmental condition in childhood, characterized by a complex pathogenesis involving the interplay of dopaminergic and serotonergic neurotransmitter imbalances, immune-inflammatory disturbances, genetic predispositions, and environmental factors (Ueda et al., 2021). Despite these insights, the precise molecular mechanisms underlying the disorder remain only partially understood. Clinically, tic disorder continues to present challenges, including a relatively high incidence of misdiagnosis and varied treatment responses (Johnson et al., 2023). Recent research has increasingly focused on the role of trace elements and vitamin D, given their critical functions in neurotransmitter synthesis, synaptic plasticity, oxidative stress regulation, and central immune homeostasis (Ma et al., 2025; Chaudhary et al., 2024). Disruptions in the homeostasis of these elements have been implicated in neurodevelopmental disorders. This study employs liquid chromatography-tandem mass spectrometry and inductively coupled plasma-mass spectrometry to systematically analyze the serum profiles of 25(OH)D and 10 trace elements in children diagnosed with tic disorder in Hubei Province. The objective is to provide empirical evidence for identifying nutritional metabolic targets and facilitating early intervention strategies for tic disorder.

The present study identified a significantly greater male predominance and a notably higher median age at onset among children in the TD group compared to the control group, aligning with prior epidemiological research (Wang et al., 2025). These observed disparities may be attributed to the modulatory effects of sex hormones on the central nervous system. Specifically, increased testosterone levels during male puberty may intensify dysregulation of dopaminergic signaling within the striatum, thereby heightening vulnerability to tic disorders. We conducted additional research on the relationships between common trace elements and 25(OH)D in children with TD. The findings showed that 25(OH)D had the strongest correlations with calcium and copper in these children, with notable correlations also observed among various trace elements. The results imply that there might be synergistic or antagonistic interactions between trace elements and vitamin D in the development and progression of TD, which together affect the stability of the nervous system.

Between-group comparisons revealed that serum 25(OH)D levels were significantly lower in children with TD, indicating a potential correlation between 25(OH)D and TD. Logistic regression models, both univariate and multivariate, were developed to examine the link between serum 25(OH)D levels and TD risk, adjusting for age and sex as confounding variables. The results demonstrated that elevated 25(OH)D levels were significantly associated with a reduced risk of TD, regardless of adjustments for age and sex, suggesting that 25(OH)D may serve as a protective factor against TD. As a neuroactive steroid hormone, vitamin D is crucial for brain development, neuroimmune regulation, and neurotransmitter synthesis. This study corroborated previous findings by demonstrating that serum 25(OH)D levels were significantly lower in children with TD compared to healthy controls, and its deficiency was identified as an independent risk factor for TD, consistent with several prior studies (Zhu et al., 2024). The mechanism potentially involves vitamin D, through its nuclear receptor, in the facilitation of neurotransmitter release, modulation of cellular calcium signaling, and regulation of antioxidant activity. This process influences the expression of neurotrophic factors such as glial cell line-derived neurotrophic factor (GDNF) and affects the balance of dopamine and serotonin systems, thereby exerting anti-inflammatory and antioxidant effects (Geng et al., 2025). A deficiency in 25(OH)D may disrupt these physiological processes, impair central nervous system function, and elevate the risk of TD. An age-stratified subgroup analysis revealed that among children aged ≤6 years, no significant differences were observed in any other indicators except for cadmium. Conversely, for those aged > 6 years, 25(OH)D levels were significantly lower in the TD group compared to controls. This finding suggests that the school-age period constitutes a critical window during which nutritional and metabolic abnormalities may contribute to the onset of TD. Children within this age range experience accelerated growth and heightened nutritional requirements, which, when combined with decreased outdoor activity and insufficient sunlight exposure for vitamin D synthesis, exacerbate deficiencies in 25(OH)D and essential trace elements (Kumar et al., 2022). Additionally, as the nervous system undergoes maturation, the adverse effects of nutritional imbalances on neural circuits are more likely to manifest clinically as tic symptoms.

Calcium and copper are crucial for maintaining normal neurological function. This study identified reduced levels of calcium and copper in the TD group, with levels in children with TD over 6 years of age being significantly lower than those in children aged 6 years or younger. This finding suggests a potential involvement of calcium and copper deficiency in the pathogenesis of TD. Calcium plays a role in regulating neuronal excitability and synaptic transmission, whereas copper serves as an essential cofactor for various neurotransmitter synthetases, such as cytochrome C oxidase and dopamine β-hydroxylase. A deficiency in copper may disrupt the metabolic balance of dopamine and norepinephrine, leading to neurotransmitter dysregulation and, consequently, tic symptoms (Lane et al., 2025; Li et al., 2025). Iron, another essential trace element, is involved in hemoglobin synthesis and neurotransmitter metabolism, including dopamine (Gao et al., 2025). In contrast to some studies that associate iron deficiency with neurodevelopmental disorders, our study found elevated iron levels in the TD group (Qian et al., 2019). The observed discrepancy may be attributable to dietary patterns specific to Hubei or variations in the regulation of iron metabolism. Excessive iron can induce oxidative stress by generating reactive oxygen species, which subsequently damage neuronal cell membranes and impair central nervous system function—a mechanism that warrants further investigation. Notably, serum ferritin, a sensitive indicator of systemic iron metabolism, was not assessed in our study. Future research should consider incorporating this parameter to elucidate the relationship between iron levels and the development of TD. Additionally, cadmium, a neurotoxic heavy metal, poses a direct threat to neuronal integrity upon accumulation. Our analysis indicated that cadmium concentrations were significantly elevated in the TD group compared to controls, with a more pronounced disparity observed in the subgroup aged over 6 years. This suggests that prolonged exposure to low doses of cadmium may elevate the risk of TD. Cadmium is capable of crossing the blood–brain barrier, accumulating in brain tissue, impairing mitochondrial function, disrupting neurotransmitter release, and inducing inflammatory responses, thereby altering the microenvironment of the central nervous system (Arruebarrena et al., 2023). This pathway may represent a critical mechanism by which cadmium contributes to the pathogenesis of TD.

Stratification by the Yale Global Tic Severity Scale (YGTSS) demonstrated no significant differences in 25(OH)D or trace element levels between children with mild TD and those with moderate-to-severe TD. Only age was higher in the moderate-to-severe group than in the mild group. These findings suggest that abnormalities in 25(OH)D and specific trace elements may contribute to the susceptibility or threshold mechanism of TD, rather than directly affecting tic intensity or frequency, and are not clearly linked to disease severity. The severity of TD may be more significantly influenced by factors such as genetic predisposition, psychological stress, and comorbidities, whereas trace elements and vitamin D are primarily implicated in the initial stages of the disease.

## 5 Study limitations

The study’s strengths include a relatively large sample size, the use of precise detection methods (LC–MS/MS and ICP-MS), and a detailed age-stratified analysis. However, several limitations should be acknowledged. First, this was a single-center retrospective study with age-mismatched cohorts, and the sample was restricted to the Hubei region, which may introduce geographical selection bias. Second, detailed information on dietary structure, outdoor activity time, and environmental exposure history was not collected, precluding the exclusion of potential confounding effects of these factors on the measured biomarkers. Furthermore, as a cross-sectional study lacking longitudinal follow-up, it is unable to establish causal relationships between changes in trace element or 25(OH)D levels and the progression of TD. Future research should involve multicenter, matched-design prospective studies with larger sample sizes, comprehensive nutritional assessments (e.g., BMI percentiles, dietary surveys) and more controlled confounding variables, in conjunction with animal experiments, to elucidate the underlying mechanisms.

## 6 Conclusion

In summary, our study indicates that children with TD in Hubei present a distinct micronutrient profile disturbance characterized by 25(OH)D deficiency, decreased calcium and copper, and increased iron and cadmium, with a more pronounced association observed in boys over the age of six. Serum 25(OH)D levels emerge as an independent protective factor against TD, particularly in school-aged children, offering potential new directions for clinical practice. Routine screening for vitamin D and key trace elements in children suspected or diagnosed with TD is recommended as a valuable reference. Timely interventions addressing abnormalities, such as vitamin D and calcium supplementation, dietary modifications to mitigate iron overload, and the avoidance of cadmium exposure, may contribute to reducing the risk of tic disorders or alleviating symptoms. Additionally, enhanced nutritional monitoring and health management for high-risk groups, such as boys over the age of six, could facilitate early prevention of TD. Both deficiencies and excesses of nutritional elements might serve as valuable and stable diagnostic markers for the early identification of tics. The differences observed in this context may also provide opportunities for the development of precision medicine approaches, leading to targeted support for learning, cognition, and daily living activities. Although the efficacy of vitamin D supplementation in improving tic symptoms requires validation through large-scale prospective intervention trials, maintaining adequate vitamin D levels and balanced mineral nutrition is consistent with general principles of neurodevelopmental health. To enhance the understanding of TD, future studies should track the dynamic changes in these indicators over time and explore their links with particular genetic variations, neuroimaging features, and treatment responses.

## Data Availability

The original contributions presented in the study are included in the article/supplementary material, further inquiries can be directed to the corresponding author.
